# The Combination of Marketed Antagonists of α1b-Adrenergic and 5-HT2A Receptors Inhibits Behavioral Sensitization and Preference to Alcohol in Mice: A Promising Approach for the Treatment of Alcohol Dependence

**DOI:** 10.1371/journal.pone.0151242

**Published:** 2016-03-11

**Authors:** Fabrice Trovero, Sabrina David, Philippe Bernard, Alain Puech, Jean-Charles Bizot, Jean-Pol Tassin

**Affiliations:** 1 Key-Obs SAS, Orléans, France; 2 Greenpharma SAS, Orléans, France; 3 Service de pharmacologie, groupe hospitalier Pitié-Salpêtrière, Paris, France; 4 Sorbonne Universités, Université Pierre et Marie Curie (UPMC) Paris 06, Institut de Biologie Paris Seine (IBPS), UM119 Neuroscience Paris Seine, Paris, France; 5 Institut National de la Santé et de la Recherche Médicale (INSERM), UMR-S 1130, Neuroscience Paris Seine, Paris, France; 6 Centre National de la Recherche Scientifique (CNRS) UMR 8246, Neuroscience Paris Seine, Paris, France; Nathan Kline Institute for Psychiatric Research and New York School of Medicine, UNITED STATES

## Abstract

Alcohol-dependence is a chronic disease with a dramatic and expensive social impact. Previous studies have indicated that the blockade of two monoaminergic receptors, α1b-adrenergic and 5-HT2A, could inhibit the development of behavioral sensitization to drugs of abuse, a hallmark of drug-seeking and drug-taking behaviors in rodents. Here, in order to develop a potential therapeutic treatment of alcohol dependence in humans, we have blocked these two monoaminergic receptors by a combination of antagonists already approved by Health Agencies. We show that the association of ifenprodil (1 mg/kg) and cyproheptadine (1 mg/kg) (α1-adrenergic and 5-HT2 receptor antagonists marketed as Vadilex ® and Periactine ® in France, respectively) blocks behavioral sensitization to amphetamine in C57Bl6 mice and to alcohol in DBA2 mice. Moreover, this combination of antagonists inhibits alcohol intake in mice habituated to alcohol (10% v/v) and reverses their alcohol preference. Finally, in order to verify that the effect of ifenprodil was not due to its anti-NMDA receptors property, we have shown that a combination of prazosin (0.5 mg/kg, an α1b-adrenergic antagonist, Mini-Press ® in France) and cyproheptadine (1 mg/kg) could also reverse alcohol preference. Altogether these findings strongly suggest that combined prazosin and cyproheptadine could be efficient as a therapy to treat alcoholism in humans. Finally, because α1b-adrenergic and 5-HT2A receptors blockade also inhibits behavioral sensitization to psychostimulants, opioids and tobacco, it cannot be excluded that this combination will exhibit some efficacy in the treatment of addiction to other abused drugs.

## Introduction

Alcoholism has been recognized as a chronic disease with a dramatic and expensive social impact [1]. However, and despite intensive research, the neurobiological substrates responsible for this disease are still poorly understood.

Ethanol is a small amphiphilic molecule which can bind to numerous sites in the central nervous system such as cell membranes and their receptors. For example, ethanol was shown to inhibit NMDA-mediated glutamatergic transmission [2] and to potentiate GABAergic inhibitory currents [3]. Ethanol was also shown to increase dopamine release in rat nucleus accumbens [4], a property which is shared by most drugs abused by humans, such as psychostimulants and opiates. Also like psychostimulants and opiates, ethanol can trigger in rodents a locomotor response that increases with repeated injections, especially in DBA/2J or Swiss (CD1) mice [5–8]. This phenomenon, called behavioral sensitization, is thought to play a critical role in the development of drug-taking and drug-seeking behaviors [9–11].

Since 2006, we have shown that the development and the expression of behavioral sensitization to most drugs of abuse were under the control of two monoaminergic receptors, the α1b-adrenergic and 5-HT2A receptors [12–15]. This led us to propose that the repeated consumption of drugs of abuse could, through repeated activations of noradrenergic and/or serotonergic neurons, uncouple a mutual control between the two systems [12, 16]. In line with this concept, we made the hypothesis that repeated treatments with specific antagonists of both noradrenergic and serotonergic receptors may facilitate a re-coupling of these systems and, thus, possibly intervene on withdrawal and relapse.

To test this hypothesis we have used antagonists of α1b-adrenergic and 5-HT2A receptors approved by Health Agencies to treat repeatedly mice previously sensitized to drugs of abuse. Because amphetamine is known to trigger a potent behavioral sensitization [10, 12, 17], we have first tested these antagonists on amphetamine-induced sensitization. Then, same experiments were performed with alcohol. Finally, since experiments indicated that these antagonists could block behavioral sensitization to alcohol, we have further examined their effects on alcohol consumption and its preference.

## Materials and Methods

### Animals and drugs

Male C57BL/6J and DBA2 mice from Janvier (Le Genest St Isle, France) were used for the experiments. Animals arrived at 4 to 6 weeks of age and were 9 weeks-old at the beginning of the experiments. They were housed 4 per cage in a regulated environment (temperature: 22 ± 1°C, 12:12 h light/dark cycle, light on at 08:00 h, light/dark cycle is inversed for alcohol preference studies where) with food and water freely available.

The experiments were conducted in agreement with the institutional guidelines for use of animals and their care, in compliance with national and international laws and policies (council directives no. 87–848, October 19, 1987, Ministere de l’Agriculture et de la Foret, Service Veterinaire de la Sante et de la Protection Animale—Key-Obs’ agreement n° A 45-234-8, permission no. 45–15 J.C.B.). Mice were sacrificed by cervical dislocation. The experimental protocols and euthanasia have been approved by the Ethics Committee of Animal experimentation, registered number 27 at the French Ministry of Research. All efforts were made to minimize the number of animals used and their suffering.

All drugs (ifenprodil tartrate, cyproheptadine hydrochloride, d-amphetamine sulfate, prazosin hydrochloride), have been purchased at Sigma (France) and were injected intraperitoneally, doses being indicated as salts.

### Amphetamine-induced behavioral sensitization

The animal (C57BL/6J mouse) is placed in an open field which it can explore freely. The activity of the animal is evaluated by the traversed total distance. Locomotor activity is registered per 5 min-periods during 90 min-session. At t = 15 min animals receive the first injection (saline, ifenprodil, cyproheptadine or ifenprodil + cyproheptadine). At t = 30 min, *i*.*e*. 30 min after the introduction of the animals in the open field, they received the 2nd injection (saline or d-amphetamine).

### Alcohol-induced behavioral sensitization

To test behavioral sensitization to alcohol we used DBA/ 2J male mice, 4–6 weeks-old at the arrival in the laboratory and weighing 21 to 27 grams at the beginning of the experiments. They are maintained 6 per cage, on a 12-h light/dark cycle (lights on at 07.00 a.m.) with food and water freely available.

The animal is placed for a 10-min session in an open field (42 × 42× 40 cm) which it can explore freely. The distance travelled is automatically measured with an infrared photobeam detection system (software Acti-track®—Panlab S.I., Bioseb, France).

Alcohol is dissolved in water solution (10% v/v) and injected intraperitoneally at the dose of 1 g/kg (injection volume: 10 ml/kg) and placed in the open field for the actimetry session. Ifenprodil and cyproheptadine are dissolved in 0.9% NaCl in order to obtain a 10 ml/kg final injection volume. The solutions were prepared the day of the experiment and administered 30 min before the alcohol administration. Animals were submitted to three sessions of habituation and received two saline administrations 30 and at the beginning of the open field session. They were then exposed to alcohol administration for sensitization sessions.

### Alcohol consumption

Animals (C57BL/6J mice) were housed in individual cages with food and water freely available. They were exposed for a two hour period from 7.00 pm to 9.00 pm (light off) to a choice protocol adapted from our colleagues [18–20].

Two bottles containing either 10% ethanol (v/v) or water were used. The preference index Alcohol/Water (Pref.) is calculated as follows: Pref. = (Alcohol consumption–water consumption) / (Alcohol consumption + water consumption) Solutions of prazosin, ifenprodil and cyproheptadine were injected in a volume of 10ml/kg of body weight 30 min before the alcohol preference session.

### Statistical analysis

Statistical evaluation of differences between the experimental groups were determined by using parametric tests, Student’s t-test or one-way or repeated measures ANOVA followed in case of p<0.05 by a Bonferroni/Dunns or Dunnet post-hoc test as appropriate.

## Results

### Effects of ifenprodil and cyproheptadine on amphetamine-induced behavioral sensitization

Previous experiments have indicated that the specific blockade of α1b-adrenergic and 5-HT2A receptors could block behavioral sensitization to amphetamine [12, 21]. Ifenprodil and cyproheptadine possesses α1-adrenergic and 5-HT2 antagonist properties, respectively [22, 23]. We have chosen these two compounds because, although they are not specific for these receptors, they are good antagonists of these receptors and have been approved by Health agencies and thus on market. Their trade names in France are Vadilex ® and Periactine ®, respectively.

**Fig 1:**
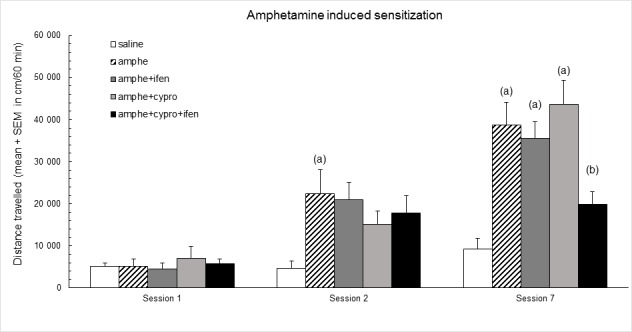
Effect of ifenprodil and cyproheptadine treatments on the d-amphetamine-induced sensitization. Session 1: all groups received two saline administrations, 15 min and 30 min after being placed in the actimeter. Then, all amphetamine groups are treated with amphetamine (2 mg/kg) as indicated in materials and methods. Sessions 2 and 7: groups received saline or ifenprodil 1 mg/kg or/and cyproheptadine 1 mg/kg and then saline and amphetamine, as indicated in materials and methods.—Y axis: distance travelled in cm (mean ± S.E.M.). Statistical analyses: repeated measures ANOVA, group effect F_4;35_ = 9.0, p< 0.001, session effect F_2;70_ = 63.2, p< 0.001, group × session interaction F_8;70_ = 5.2, p< 0.001; one-way ANOVAs, differences between groups in session 1 F_4;35_ = 0.2, p = ns; in session 2 F_4;35_ = 3.0, p< 0.05, in session 7 F_4;35_ = 11.0, p< 0.001; post-hoc comparisons, Bonferroni/Dunn test, (a) significantly different (*p*<0.05) from saline group in each session; (b) significantly different (*p<*0.05) from amphetamine group.

First, we have tested whether these compounds could affect behavioral sensitization to amphetamine. Fig 1 indicates that, when repeatedly injected with d-amphetamine, animals pretreated with ifenprodil and cyproheptadine do not exhibit any locomotor sensitization when compared to animals pre-treated with saline (p<0.01, session 7). Interestingly, this inhibition of amphetamine behavioral sensitization does not occur if animals are pre-treated with either ifenprodil or cyproheptadine alone, at the doses tested here (Session 7).

Moreover, it should be emphasized that, at the doses used, the combination of ifenprodil and cyproheptadine does not affect the acute amphetamine-induced locomotor hyperactivity (Fig 1, session 2).

### Effects of ifenprodil and cyproheptadine on alcohol behavioral sensitization

**Fig 2:**
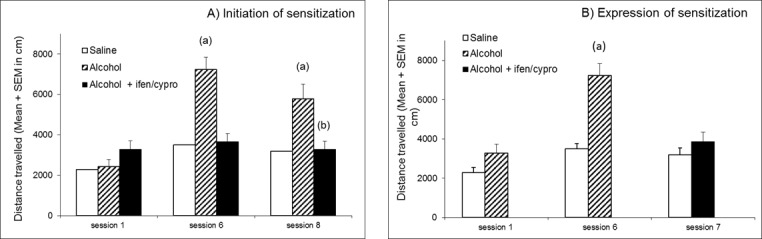
Effect of a treatment by ifenprodil + cyproheptadine in combination on alcohol induced sensitization. Y axis: distance travelled in cm (mean ± S.E.M.) A) Initiation of sensitization Session 1: all groups (N = 8 animals per group) received two saline administrations, 15 min and 30 min after being placed in the actimeter. Sessions 2 to 8: groups received saline or ifenprodil 1 mg/kg + cyproheptadine 1 mg/kg and then saline or alcohol (1g/kg ip/session), as indicated in materials and methods. Statistical analyses: repeated measures ANOVA, group effect F_2;21_ = 14.2, p< 0.001, session effect F_2;42_ = 22.6, p< 0.001, group × session interaction F_4;42_ = 9.5, p< 0.001; one-way ANOVAs, differences between groups in session 1 F_2;21_ = 2.2, p = ns; in session 6 F_2;21_ = 21.7, p< 0.001, in session 8 F_2;21_ = 8.3, p< 0.01; post-hoc comparisons, Bonferroni/Dunn test, (a) significantly different (*p*<0.05) from saline group; (b) significantly different (*p<*0.05) from alcohol group. B) Expression of sensitization: all animals (N = 8 animals per group) received alcohol and no further treatment until session 7. At session 7, animals sensitized to alcohol received ifenprodil (1 mg/kg) + cyproheptadine (1 mg/kg) before alcohol administration (1 g/kg, ip). Statistical analyses: repeated measures ANOVA, group effect F_1;14_ = 30.9, p< 0.001, session effect F_2;28_ = 19.0, p< 0.001, group × session interaction F_2;28_ = 7.6, p< 0.01; Student’s t-test, (a) significantly different (*p*<0.05) from saline group.

Because C57Bl6 mice do not exhibit any locomotor activation following alcohol treatment, DBA2 mice were used for this experiment [6, 7]. In our hands, an acute injection of alcohol induced only a tendency towards an increase of locomotor activity in DBA2 mice (Fig 2A, session 1). This locomotor response clearly increased after repeated injections of alcohol (p<0.001) (Fig 2A, session 8). Interestingly, this behavioral sensitization was not observed in animals pretreated at each session with a combination of cyproheptadine and ifenprodil (Fig 2A, session 8).

Moreover, Fig 2B shows that, after repeated treatments with alcohol and the development of a locomotor sensitization (Fig 2B, session 6), a subsequent treatment with a combination of ifenprodil and cyproheptadine completely abolishes the locomotor sensitization to alcohol (Fig 2B, session 7).

### Effects of the combination of ifenprodil and cyproheptadine on alcohol intake

**Fig 3:**
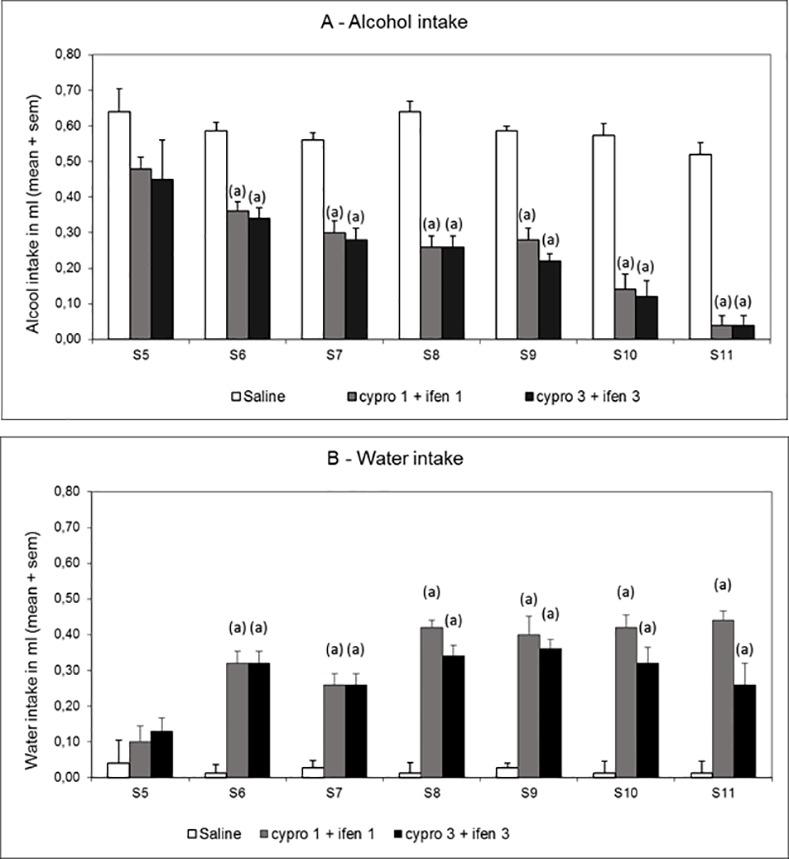
**Effect of ifenprodil (ifen) and cyproheptadine (cypro) in combination on alcohol intake (Fig 3A) and on water intake (Fig 3B) during 2 hour sessions with alcohol (10%) and water bottles.** Animals were previously exposed for 10 days to forced alcoholic consumption. Ifenprodil and cyproheptadine are administered 30 min before each alcohol session (from session 5 to session 11), at the doses of 1 mg/kg or 3 mg/kg (ifen/cypro 1/1 and ifen/cypro 3/3) (Saline group, N = 15; Cypro 1 + Ifen 1 and Cypro 3 + Ifen 3 groups, N = 10). Statistical analyses: repeated measures ANOVA, alcohol intake, group effect F_2;32_ = 299.1, p< 0.001, session effect F_7;224_ = 17.4, p< 0.001, group × session interaction F_14;224_ = 3.0, p< 0.001; water intake, group effect F_2;32_ = 108.5, p< 0.001, session effect F_7;224_ = 12.4, p< 0.001, group × session interaction F_14;224_ = 4.5, p< 0.001; one-way ANOVAs, differences between groups in session 5, alcohol intake, F_2;32_ = 2.0, p = ns; water intake, F_2;32_ = 2.1, p = ns; in sessions 6–12, F_2;32_≥ 11.0, p< 0.001; post-hoc comparisons, Dunnett test, (a) significantly different (*p*<0.05) from saline group.

In these experiments and the following ones we switched to C57Bl6 mice because of their high sensitivity to alcohol intake [24]. First, mice are exposed during 10 days at forced alcohol consumption, the only available beverage in the cage being alcohol (10% v/v). On the 11^th^ day, they are submitted to the combination of ifenprodil and cyproheptadine 30 minutes before the two hours sessions of choice between drinking water or alcohol (10% v/v) (Fig 3, session 5). The same experiment is repeated every day during 6 days (Fig 3 from session 6 to session 11).

Data indicate a progressive decreased consumption of alcohol in mice which leads to an almost complete (92%) abstinence of alcohol (Fig 3A) and to an increase in water intake (Fig 3B) indicating that the compounds do not affect general liquid intake. The effects on alcohol abstinence are significant for both doses of combined ifenprodil and cyproheptadiine (1/1 and 3/3 mg/kg) from session 6 to session 11 (p<0.01).

### Effects of the combination of ifenprodil and cyproheptadine on alcohol preference

**Fig 4:**
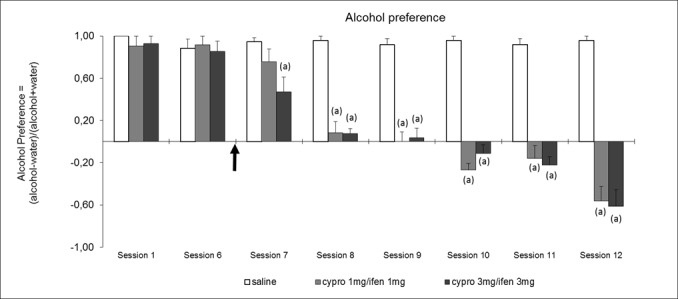
Effect of ifenprodil (ifen) and cyproheptadine (cypro) in combination on alcohol preference during 2 hour sessions with alcohol (10%) and water bottles. The preference index Alcohol/Water (Pref.) is calculated as follows: Pref. = (Alcohol consumption–water consumption) / (Alcohol consumption + water consumption). All groups (Saline, N = 12, cypro 1 + ifen 1, N = 8, cypro 3 + ifen 3, N = 9 received saline treatment at the first two sessions, cyproheptadine and ifenprodil treatments started at the arrow. Results are expressed as mean values ± S.E.M. Statistical analyses: repeated measures ANOVA, group effect F_2;26_ = 223.2, p< 0.001, session effect F_6;156_ = 45.7, p< 0.001, group × session interaction F_12;156_ = 9.5, p< 0.001; one-way ANOVAs, differences between groups in session 1 F_2;21_ = 0.7, p = ns; in session 6 F_2;26_ = 0.1, p = ns; in sessions 7–12, F_2;26_≥ 6.1, p< 0.01; post-hoc comparisons, Dunnett test, (a) significantly different (*p*<0.05) from saline group.

The data obtained on alcohol intake led us to test the same combination of compounds on alcohol preference. In this experiment animals, after having been forced to drink alcohol (10% v/v) during 10 days, have the possibility on the 11^th^ day, two hours per day, to choose between drinking water or alcohol (10% v/v). Fig 4 shows that animals attain rapidly a preference of 70% for alcohol (Sessions 1 and 2). However, when animals are pre-treated with the combination of ifenprodil and cyproheptadine, the preference for alcohol decreases gradually from session 3 to 5 and becomes an aversion from session 6 to 8. At the end of the experiment the animals exhibit a preference for water of almost 60% (p<0.01).

### Effects of the combination of prazosin and cyproheptadine on alcohol preference

As mentioned above, ifenprodil and cyproheptadine are not specific for α1-adrenergic and 5-HT2 receptors. This is particularly true for ifenprodil which is not only an α1-adrenergic receptor antagonist but also a well-known non competitive antagonist of NMDA receptors [22]. We therefore tried to test if we could obtain the same data on alcohol preference by a combination of another commercially available specific α1-adrenergic antagonist, prazosin (Mini-press ® in France).

**Fig 5:**
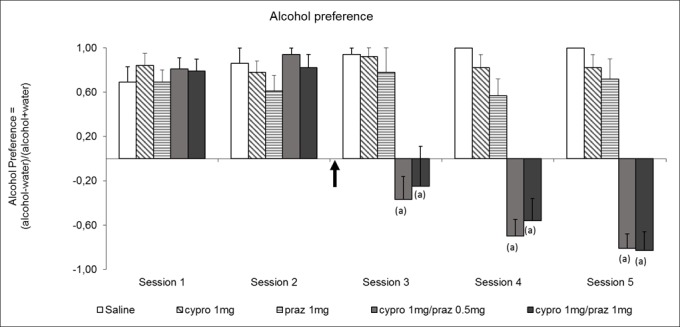
Effect of prazosin (praz) or cyproheptadine (cypro) separately or in combination on alcohol preference during 2 hour sessions with alcohol (10%) and water bottles. Doses of cyproheptadine and prazosin were 1 mg/kg and 0.5 mg/kg, respectively. The preference index Alcohol/Water (Pref.) is calculated as follows: Pref. = (Alcohol consumption–water consumption) / (Alcohol consumption + water consumption). All groups (N = 6 animals per group except cypro 1 + prazo 0.5, N = 9) received saline treatment at the first two sessions, cyproheptadine and prazosin treatments started at the arrow. Results are expressed as mean values ± S.E.M. Statistical analyses: repeated measures ANOVA, group effect F_4;28_ = 54.8, p< 0.001, session effect F_4;112_ = 18.0, p< 0.001, group × session interaction F_16;1112_ = 10.0, p< 0.001; one-way ANOVAs, differences between groups in session 1 and 2, F_4;28_≤ 1.3, p = ns; in sessions 3–5 F_4;28_≥ 9.5, p< 0.001; post-hoc comparisons, Dunnett test, (a) significantly different (*p*<0.05) from saline group.

Data indicate, as previously found with ifenprodil, that the combination of prazosin and cyproheptadine induced a progressive decrease of the alcohol preference to finally attain a clear preference for water (80%, Fig 5, session 5). This experiment also indicates that, if prazosin or cyproheptadine is given alone at the dose tested here, there is no significant effect on alcohol preference in our experimental conditions, whatever the session (Fig 5, sessions 3 to 5).

## Discussion

Numerous strategies involving specific systems of neurotransmission, have been performed to treat alcohol-dependence. Acamprosate has been tried as an antagonist of glutamatergic transmission, naltrexone and nalmefene are used as antagonists of opioid receptors and disulfiram has been selected as an inhibitor of acetaldehyde dehydrogenase and of dopamine β-hydroxylase, the last enzymatic step in the synthesis of noradrenaline. Finally, GABAergic transmission was also targeted with benzodiazepines and, more recently, with γ-hydroxy-butyrate or baclofen, an agonist of GABAb receptors [25].

Here, in line with our findings on behavioral sensitization to drugs of abuse, we have chosen to block the two monoaminergic receptors seemingly responsible for the uncoupling of noradrenergic and serotonergic neurons following repeated drugs of abuse consumption. Because our ultimate aim was to find a therapeutic treatment of alcohol dependence in humans, we have chosen to block α1b-adrenergic and 5-HT2A receptors with drugs already approved by Health Agencies and administered to patients for several years.

The main finding of our study is that the combination of two blocking agents, prazosin, an antagonist of α1b-adrenergic receptors, and cyproheptadine, an antagonist of 5-HT2 receptors, including the 5HT2A subtype, is able to almost completely reverse alcohol preference in mice (Fig 5). Moreover, when animals are treated by the same dose of each antagonist alone, no effect on alcohol preference is observed (Fig 5), strongly suggesting the presence of a cooperation or a synergy between noradrenergic and serotonergic transmissions.

Very similar data on alcohol preference have also been obtained here when ifenprodil was given instead of prazosin. This indicates that the decreased preference to alcohol observed after repeated treatments with the combination of ifenprodil and cyproheptadine (Fig 4), is not due to the antagonist properties of ifenprodil on NMDA receptors. It was crucial to verify this point because it has been demonstrated some years ago that inhibition of NMDA receptors can block the behavioral sensitization to psychomotor stimulants [17, 26]. Our data clearly suggest that it is the α1-adrenergic receptor’s blocking characteristics of ifenprodil that are responsible for the effects we observe on alcohol behavioral sensitization and preference.

The combined treatment with ifenprodil and cyproheptadine is also able to inhibit by 92% alcohol intake in animals previously forced to drink alcohol for 10 days (Fig 3). This paradigm is particularly interesting because it reflects a situation which is similar to what could occur in alcohol-dependent humans. At this state of our experiments, it cannot be determined whether animals stop drinking alcohol because they are not anymore interested or because alcohol becomes aversive. In any case, a study on the effect of a combined treatment of prazosin and cyproheptadine in alcohol-dependent patients is even more tempting that both compounds have shown their lack of toxicity after several years on drug market in different countries.

Interpretation of behavioral sensitization experiments is more complex since this phenomenon, specific for drugs of abuse, seems to occur only in rodents. We have shown that repeated treatments with drugs of abuse induce a hyper-reactivity of both noradrenergic and serotonergic neurons [12, 13], the behavioral output of this neuronal hyper-reactivity being, in rodents, an increased locomotor response when compared with that induced by an acute injection. Although behavioral sensitization cannot be observed in humans, it cannot be excluded that the hyper-reactivity of noradrenergic and serotonergic neurons is nevertheless present in human addicts. It even may be the neuronal basis of drug addiction in the sense that any emotion would induce increased activations of the two neuronal systems. Blocking the consequence of these neuronal exacerbated activations with noradrenergic and serotonergic receptors antagonists, as performed here, could potentially prevent their dysregulation, diminish the discomfort felt by human addicts and thus facilitate abstinence.

Pre-clinical studies have indicated that prazosin associated with propranolol, a β-adrenergic receptor antagonist, or naltrexone, an opioid receptor antagonist, decreases alcohol drinking more effectively than either drug alone [27–29]. Moreover, recent clinical studies have shown that prazosin could diminish the quantity of alcohol ingested by alcohol-dependent patients [30]. Although significant, this improvement was small. This indicates that the blockade of α1-adrenergic receptors is important but still not sufficient if not associated with the modification of another neuronal transmission. In this regard, the authors of the clinical trial with prazosin concluded that a combination with another compound would be appropriate to reach a robust therapeutic benefit [30]. Our data suggest that the other neuronal transmission could be serotonergic through a 5-HT2A component. Indeed, as previously mentioned, the combination of the two compounds modifies by almost 90% alcohol preference whereas the same dose of each compound has no effect by itself (Fig 5 Session 5)

Altogether our data which resulted in published patents (WO2006018538 (A1)–drug for the treatment of central nervous system disorders–date 02/23/2006 and WO 2012/175894 A1 Pharmaceutical composition for treating dependency in human beings- date 12/27/2012) strongly suggest that clinical studies performed in alcohol-dependent patients with prazosin and cyproheptadine should be considered in the very near future. Moreover, if results are positive, it cannot be excluded that this combined treatment would also be effective as a therapy for other drugs of abuse, such as tobacco, psychostimulants or opioids. In any case, if data in alcohol-dependent patients appear to be positive, they may represent an important step in the treatment of addiction to other compounds.
